# Light-Dependent Phosphorylation of the *Drosophila* Inactivation No Afterpotential D (INAD) Scaffolding Protein at Thr170 and Ser174 by Eye-Specific Protein Kinase C

**DOI:** 10.1371/journal.pone.0122039

**Published:** 2015-03-23

**Authors:** Olaf Voolstra, Philipp Spät, Claudia Oberegelsbacher, Björn Claussen, Jens Pfannstiel, Armin Huber

**Affiliations:** 1 Department of Biosensorics, Institute of Physiology, Universität Hohenheim, Stuttgart, Germany; 2 Mass Spectrometry Core Facility, Universität Hohenheim, Stuttgart, Germany; University of Western Australia, AUSTRALIA

## Abstract

*Drosophila* inactivation no afterpotential D (INAD) is a PDZ domain-containing scaffolding protein that tethers components of the phototransduction cascade to form a supramolecular signaling complex. Here, we report the identification of eight INAD phosphorylation sites using a mass spectrometry approach. PDZ1, PDZ2, and PDZ4 each harbor one phosphorylation site, three phosphorylation sites are located in the linker region between PDZ1 and 2, one site is located between PDZ2 and PDZ3, and one site is located in the N-terminal region. Using a phosphospecific antibody, we found that INAD phosphorylated at Thr170/Ser174 was located within the rhabdomeres of the photoreceptor cells, suggesting that INAD becomes phosphorylated in this cellular compartment. INAD phosphorylation at Thr170/Ser174 depends on light, the phototransduction cascade, and on eye-Protein kinase C that is attached to INAD via one of its PDZ domains.

## Introduction

Components of the *Drosophila* phototransduction cascade are tethered together by the inactivation no afterpotential D (INAD) scaffolding protein to form a supramolecular complex that is referred to as the INAD signaling complex [1–8]. INAD is composed of five PDZ domains that provide the binding sites for INAD-attached proteins of the phototransduction cascade and a larger linker region between PDZ1 and PDZ2 with a putative binding site for calmodulin [9]. INAD ligands include the transient receptor potential (TRP) ion channel, phospholipase Cβ (PLCβ), eye protein kinase C (eye-PKC) [1–4,7], neither inactivation nor afterpotential C (NINAC) [10], and retinophilin (probably through binding to NINAC) [11,12]. The photosensitive structures of the fly photoreceptors are the rhabdomeres that are composed of finger-like evaginations of the apical membrane (microvilli). Scaffolding of signaling molecules ensures proper subcellular localization, minimizes unwanted interactions, and thus enhances signaling speed and efficiency. The term “scaffolding protein” suggests a rather static role of the INAD protein. However, over 30 years ago, a retina-specific polypeptide with an apparent molecular mass of 80 kilodalton was shown to undergo a reversible light-dependent isoelectric point shift that was caused by phosphorylation [13,14]. Later, this polypeptide was identified as INAD and peptide mass fingerprints suggested phosphorylation at various positions [15]. Recently, the dynamic nature of the INAD scaffolding protein was further corroborated by the discovery of a transient light-dependent formation of a disulfide bond between Cys606 and Cys645 within PDZ5 [16]. The authors demonstrated that the redox- and light-dependent formation of this disulfide bond led to distortion of a ligand binding site. Formation of the disulfide bond was detected in illuminated wild type flies and was diminished but not completely abolished in the absence of functional rhodopsin and eye-PKC. The latter finding led the authors to propose that INAD phosphorylation might be the signal for the formation of the disulfide bond within PDZ5. A fly transgenically expressing INAD in which Cys645 was substituted with Ser preventing formation of the disulfide bond within PDZ5, displayed a shortened refractory period of the microscopic light response being in line with the proposition that the ligand binding site within PDZ5 is permanently intact in this fly. In addition to the redox-dependent disulfide bond formation within PDZ5, Liu and coworkers established an interaction between PDZ4 and PDZ5 resulting in an elevation of the redox potential trapping the cysteines within PDZ5 in their reduced state [17]. Acidification that might be induced by PIP_2_ hydrolysis in the light by PLCβ [18], obstructs the interaction between PDZ4 and PDZ5, causing cysteine oxidation and generation of the disulfide bond within PDZ5 which in turn leads to the distortion of the ligand binding site. The interaction between PDZ4 and PDZ5 depends on formation of a hydrogen bond between His547 and Thr669. A recombinantly-expressed fragment comprising PDZ4 and 5 was strongly phosphorylated in an *in vitro* kinase assay using protein kinase C only when Thr669 was substituted by Ala providing further evidence for a possible link between phosphorylation and formation of the disulfide bond within PDZ5. The authors propose the transient receptor potential (TRP) ion channel as the ligand of INAD PDZ5. They also provide evidence that TRP additionally binds to INAD PDZ3. According to their model, TRP is released from PDZ5 in the light and is eventually released from PDZ3 as well. A fly transgenically expressing an INAD protein in which Thr669 was substituted with Ala disrupting the hydrogen bond between PDZ4 and PDZ5, displayed prolonged activation kinetics of the macroscopic light response that was caused by an increase in quantum bump latency. Additionally, this fly displayed a prolonged refractory period being in line with the proposition that PDZ5 is locked in the oxidized state and thus is uncoupled from TRP.

The current evidence outlined above suggests that light-dependent phosphorylation of INAD might contribute to the dynamic molecular rearrangements of this scaffolding protein. However, the INAD phosphorylation sites that might regulate the distortion of the ligand binding pocket within PDZ5 are not known. Here, we report the identification of eight INAD phosphorylation sites in a mass spectrometry approach. We show that phosphorylation of two of these sites, Thr170/Ser174, depends on light, the phototransduction cascade, and eye-PKC. Light-independent elevation of intracellular Ca^2+^ levels was sufficient to trigger Thr170/Ser174 phosphorylation.

## Materials and Methods

### Ethics Statement

Immunization of rabbits was conducted at the University of Karlsruhe according to the animal protection ordinance and was approved by the regional administrative council Karlsruhe (reference number 35-9158.82/977/99). All efforts were made to ensure animal welfare and minimize suffering.

### Fly Stocks

The following *Drosophila melanogaster* strains and mutants were used: w Oregon R, yw;*inaD* ^*1*^ [7], yw;;*trp* ^*P343*^ [19], w;;*trp* ^*P365*^ [20], yw;;*ninaE* ^*17*^ [21], w;*Gqα* ^*1*^ [22], w,*norpA* ^*P24*^ [23], w;*inaC* ^*P209*^ [24], w;;*trp* ^*D621G*^,*trp* ^*P343*^ [25]. Flies were reared at 25°C. 1–5 day-old-flies were used for the experiments.

For analysis of the INAD phosphorylation state, flies were illuminated with a white 18-watt fluorescent lamp, 2400 lux, or kept in the dark, for 12–18 h before they were subjected to protein extraction for 1D or 2D gel electrophoresis and subsequent Western blot analyses or to immunoprecipitation and subsequent mass spectrometry analysis. For 1D gel electrophoresis prior to Western blot analysis, flies were manually decapitated and extracts were prepared as described previously [26]. For 2D gel electrophoresis and immunoprecipitation, fly heads were obtained as described previously [26]. Briefly, flies were frozen in liquid nitrogen and vigorously vortexed. Heads were passed through a 45-mesh sieve holding back the bodies and were collected on a 25-mesh sieve (Neolab). Dark-kept flies were prepared under dim red light (Schott RG 665), whereas flies kept in white light were prepared under this light condition.

### Antibodies

To generate a phosphospecific antibody against INAD phosphorylated at pThr170 and pSer174, a phosphopeptide, NH2-T(pT)FTA(pS)MRQC-CONH2, comprising pThr170 and pSer174 was synthesized, coupled to keyhole limpet haemocyanin, and injected into rabbits. The first immunization was accomplished using Freund’s complete adjuvant and for concomitant boosts, Freund’s incomplete adjuvant was used. Peptide synthesis and immunization was done by Pineda Antibody Service. Affinity purification was carried out as described previously [26]. For immunoprecipitation of the INAD signaling complex, a polyclonal α-TRP antibody was used that was raised against amino acids 906–1275 [24]. To generate a pan-specific α-INAD antibody, a rabbit was immunized with a recombinantly-expressed polypeptide comprising amino acids 281–550. Antisera were cleaned up using a HiTrap protein A HP column (GE Healthcare Life Sciences) according to the manufacturer’s protocol. This antibody was used for Western blot analyses and immunoprecipitations. For immunocytochemistry, an α-INAD antibody was used that was kindly provided by Susan Tsunoda [7].

### 2D Gel Electrophoresis

Approximately 300 fly heads were homogenized in 200 μl 2D lysis buffer (7 M urea, 2 M thiourea, 4% CHAPS, 30 mM Tris-HCl, pH 8.5) using a micropestle. Homogenates were extracted on ice for 30 min and were then centrifuged for 10 min at 12,000 × g at 4°C. The resulting supernatant was used for 2D gel electrophoresis. Protein concentrations were determined using the Rotiquant Bradford assay (Roth). For *in vitro* dephosphorylation, head extract (600 μg of protein) was diluted into a total volume of 210 μl of the supplied buffer supplemented with 2 mM MnCl_2_, a cocktail of protease inhibitors (1 μg/μl aprotinin, 10 mM benzamidin, 2 μg/μl leupeptin, 0,01 μg/μl pepstatin A, 50 μM APMSF), and 2 μl of lambda phosphatase (New England Biolabs). Samples were then incubated at 30°C for 60 min. 600 μg of protein were cup-loaded onto 18 cm IPG strips with a linear pH gradient of 6–11 (GE Healthcare) and proteins were subjected to isoelectric focusing in an IPGphor3 system (GE Healthcare) using the following parameters: 2 h, 150 V, step; 2 h, 300 V, step; 8 h, 1000 V, gradient; 3 h, 8000 V, gradient; 5 h, 8000 V, step; 50 V, step until processing. The current was limited to 50 μA per strip. After isoelectric focusing, the IPG strips were equilibrated in equilibration buffer (6 M urea, 72 mM Tris-HCl, pH 8.8, 29.3% (v/v) glycerol, 2% SDS) containing 1% dithiothreitol and then in equilibration buffer containing 2.5% iodoacetamide 15 min each. The strips were applied to a 26 cm × 20 cm 12% SDS polyacrylamide gel and overlaid with 0.5% agarose containing 0.01% (w/v) bromphenol blue. A small piece of filter paper was soaked with Prestained protein marker (Bio-Rad) and was placed beside the strip. Gel electrophoresis was performed at an initial current of 10 mA per gel for 1 h and then at 12 mA per gel over night at 20°C. Gels were then subjected to Western blot analysis or were stained with colloidal Coomassie brilliant blue to recover protein for mass spectrometry.

### Immunoprecipitation of INAD and TRP

Immunoprecipitations of INAD and TRP were performed as described previously [26]. The INAD signaling complex from *Drosophila* head extracts was immunoprecipitated using 3 μg of α-INAD or α-TRP antibodies.

### Mass Spectrometry Analyses

Prior to nano-LC-ESI-MS analysis, INAD was in-gel digested with trypsin. Mass spectrometry was performed on a Thermo LTQ-Orbitrap instrument (Thermo) as described before [26], with modifications. Survey spectra (m/z = 300–1800) were detected in the Orbitrap at a resolution of 60,000 at m/z = 400. Phosphopeptides were identified by database searches using the MASCOT search algorithm as described in [23]. Assignment of phosphorylation sites was verified by manual inspection of MS/MS spectra.

### Western Blot Analysis

Protein transfer onto PVDF membranes (Bio-Rad) was carried out as described previously [26]. Primary antibodies used were α-INAD and α-pThr170/pSer174. α-rabbit IgG coupled to horse radish peroxidase (Sigma) was used as secondary antibody. The signal was acquired as described previously [27] using a Chemidoc XRS+ documentation system (Bio-Rad).

### Immunocytochemistry of Fly Eyes

Immunocytochemistry was carried out as described previously [28], with modifications. An α-INAD antibody [7] and an α-pThr170/pSer174 antibody were used as primary antibodies. Secondary antibodies were α-rabbit Cy5 (Dianova) and AlexaFluor 546-coupled phalloidin (Life Technologies). Images were acquired with an AxioImager.Z1m microscope (objective EC Plan-Neofluar x40/1.3 oil, Zeiss) with the ApoTome module (Zeiss) and an AxioCam MRm (Zeiss).

## Results

### The INAD Scaffolding Protein is Light-Dependently Phosphorylated

**Fig 1:**
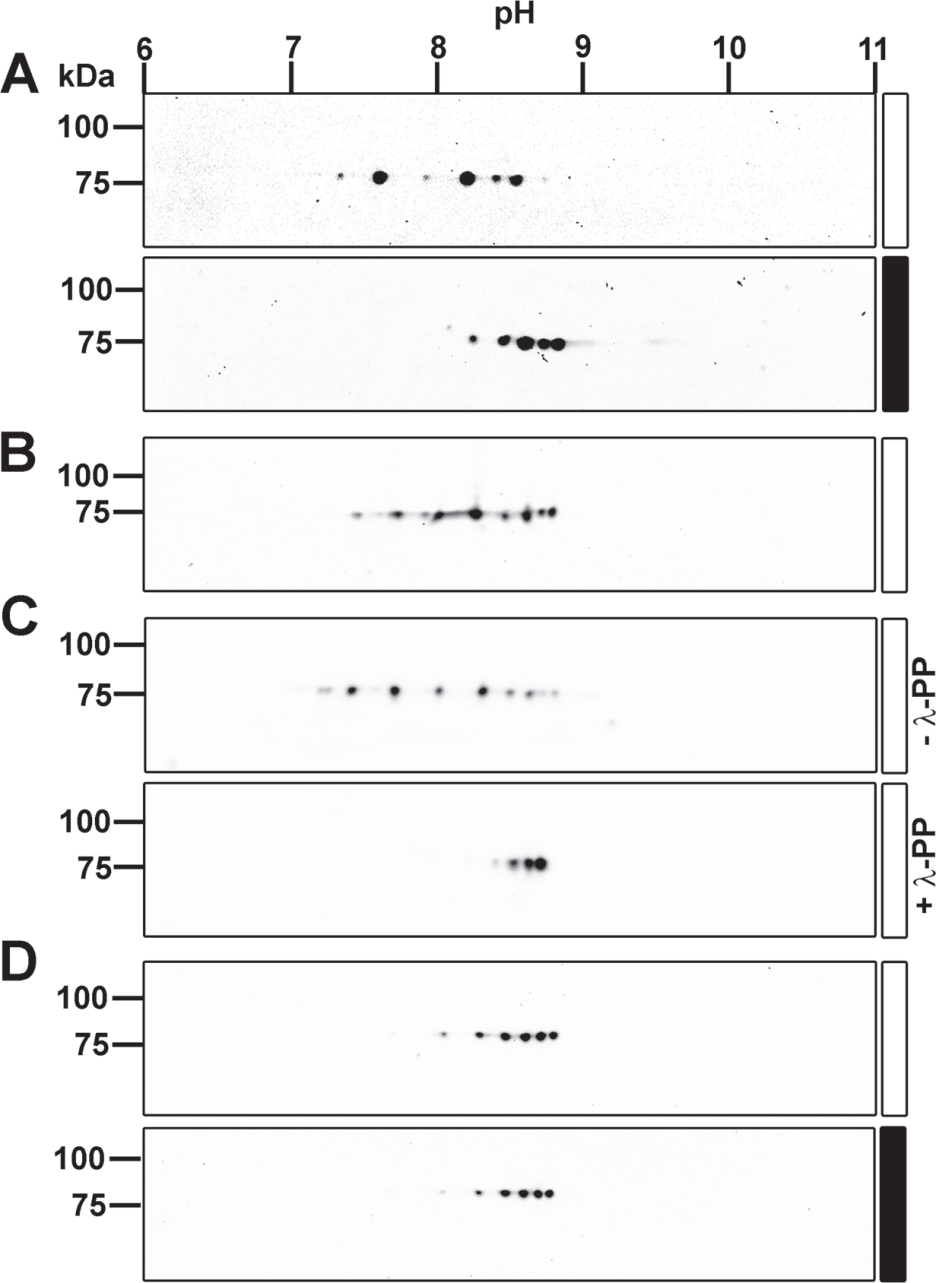
Light-Dependent Isoelectric Point Shift of the INAD Scaffolding Protein. *Drosophila* head extracts were two-dimensionally separated by isoelectric focusing and SDS gel electrophoresis, blotted onto PVDF membranes and probed with a pan-specific α-INAD antibody. A, Wild type flies were light or dark adapted for 12–18 h prior to protein extraction from heads as indicated by the bars beside the blots (white bar, 12–18 h light; black bar, 12–18 h dark). B, Wild type flies were dark adapted for 12–18 h and then illuminated for 5 seconds prior to protein extraction from heads (white bar, 5 seconds light). C, Wild type flies were light adapted for 12–18 h prior to protein extraction from heads. For λ-phosphatase treatment, protein extracts from fly heads were incubated with λ-phosphatase (+λ-PP) for 1 h at 30°C (lower panel). A sample without λ-phosphatase (-λ-PP) was carried along as a control (upper panel). D, *norpA* ^*P24*^ mutant flies lacking phospholipase Cβ were light (white bar) or dark (black bar) adapted for 12–18 h prior to protein extraction from heads. Molecular mass markers (in kDa) are indicated on the left, pH on the top.

INAD has been shown to undergo transient light-dependent phosphorylation [13–15]. To investigate light-dependent posttranslational modifications of the INAD protein in further detail, we either light or dark-adapted flies over night and subjected head extracts to 2D gel electrophoresis and immunoblotting using an α-INAD antibody (Fig. 1A). INAD proteoforms from dark-adapted wild type flies were isoelectrically focused in several protein spots in a range between pH 8 and 9. In contrast, in illuminated flies, INAD isoforms focused in the basic pH range disappeared and isoforms in the range between pH 7.5 and 8.5 appeared. To analyze the isoelectric point shift of INAD at a shorter time scale, we illuminated dark-adapted wild type flies for 5 seconds. As a result, additional proteoforms in the more acidic range became observable (Fig. 1B), although the amount was reduced as compared to light-adapted flies (see Fig. 1A). Since phosphorylation leads to a shift of the isoelectric point (pI) of proteins towards more acidic pH ranges, we concluded that INAD becomes phosphorylated in light-adapted flies. Indeed, treatment of head extracts from light-adapted flies with λ-phosphatase caused a pI shift of the INAD isoforms toward more basic pH ranges (Fig. 1C, lower panel). Omission of the phosphatase (Fig. 1C, upper panel) resulted in a spot pattern that was similar to untreated light-adapted flies (see Fig. 1A). Moreover, phosphatase treatment led to an even more condensed spot pattern in the basic range than dark adaptation indicating that INAD contains sites that are already phosphorylated in the dark. However, phosphatase treatment resulted in three remaining prominent spots suggesting the existence of other post-translational modifications besides phosphorylation. In contrast to the wild type, the spot patterns of light- and dark-adapted *norpA* ^*P24*^ mutant flies lacking the central effector enzyme of the phototransduction cascade, phospholipase Cβ are similar (Fig. 1D) and are reminiscent of the spot pattern of dark-adapted wild type flies (see Fig. 1A). We conclude that phospholipase Cβ is essential to trigger light-dependent phosphorylation of INAD indicating that this process depends on the phototransduction cascade.

### Identification of INAD phosphorylation sites

**Fig 2:**
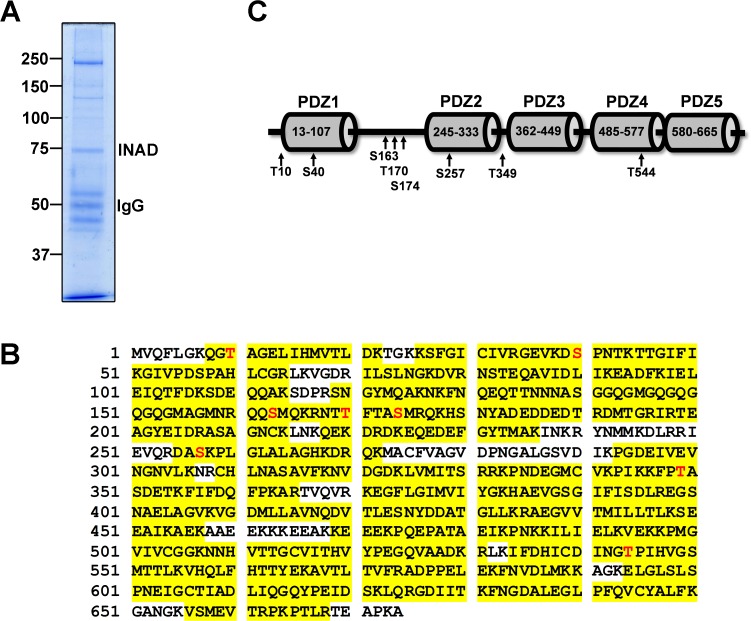
Identification of INAD Phosphorylation Sites. A, Representative Coomassie-stained polyacrylamide gel of protein extracts from wild type fly heads that were immunoprecipitated with an α-INAD antibody. The INAD band at ca. 75 kDa was excised, digested with trypsin, and subjected to nanoLC-ESI mass spectrometry. MS/MS spectra of identified phosphopeptides are shown in S2 B Fig., Cartoon of the INAD protein structure showing the positions of PDZ domains (modified from [7]) and the identified phosphorylation sites. C, INAD amino acid sequence depicting peptides that were identified by mass spectrometry (85.9% sequence coverage, highlighted in yellow) and identified phosphorylation sites indicated by red letters.

After confirmation of light-dependent phosphorylation of INAD, we set out to identify *in vivo* INAD phosphorylation sites in a mass spectrometry approach. INAD was either enriched by immunoprecipitation using α-TRP or α-INAD antibodies or separated by 2D gel electrophoreses. Excised gel bands from immunoprecipitations followed by 1D polyacrylamide gel electrophoresis or spots from 2D gel electrophoresis were in-gel digested using trypsin. A representative 1D SDS polyacrylamide gel of an immunoprecipitation using an α-INAD antibody is shown in Fig. 2A. Resulting peptides were separated by HPLC and analyzed by mass spectrometry. In total, we obtained a sequence coverage of 85.9% of the INAD protein (Fig. 2B). Collectively, eight INAD phosphorylation sites were unambiguously identified by MS/MS spectra (Fig. 2C, S1 Fig.). INAD phosphorylation sites Thr10, Ser163, Ser257, and Thr544 were identified from the 75 kDa gel band after immunoprecipitation of head extracts from light-adapted flies. While Thr10 and Ser163 were found in immunoprecipitates using an α-TRP antibody, Ser257 and Thr544 were identified in immunoprecipitates using an α-INAD antibody. Thr170 and Ser174 were identified from spots that were excised from a 2D gel using head extracts from light-adapted wild type flies. Ser174 was also found in immunoprecipitations using an α-TRP and α-INAD antibody, respectively. The phosphorylation site Ser40 was identified from an immunoprecipitation of dark-adapted *inaC* ^*P209*^ flies and the Thr349 site was identified from an immunoprecipitation of light-adapted *inaC* ^*P209*^ flies. In both cases, an α-TRP antibody was used for immunoprecipitation. Taken together, one phosphorylation site, Ser40, was identified in extracts of dark-adapted flies, and two phosphorylation sites, Ser40 and Thr349, were identified in extracts of *inaC* ^*P209*^ flies whereas the remaining sites were identified in extracts of light-adapted wild type flies. The positions of the identified INAD phosphorylation sites in relation to the PDZ domains are indicated in Fig. 2C. Whereas Thr10 is located in the N-terminal portion of INAD, Ser40, Ser257, and Thr544 reside within PDZ1, PDZ2, and PDZ4, respectively. Ser163, Thr170, and Ser174 are located in the linker region between PDZ1 and PDZ2 that harbors a putative calmodulin binding site [9]. Thr349 is located between PDZ2 and PDZ3.

### INAD-pThr170/pSer174 is Located within the Rhabdomeres

**Fig 3:**
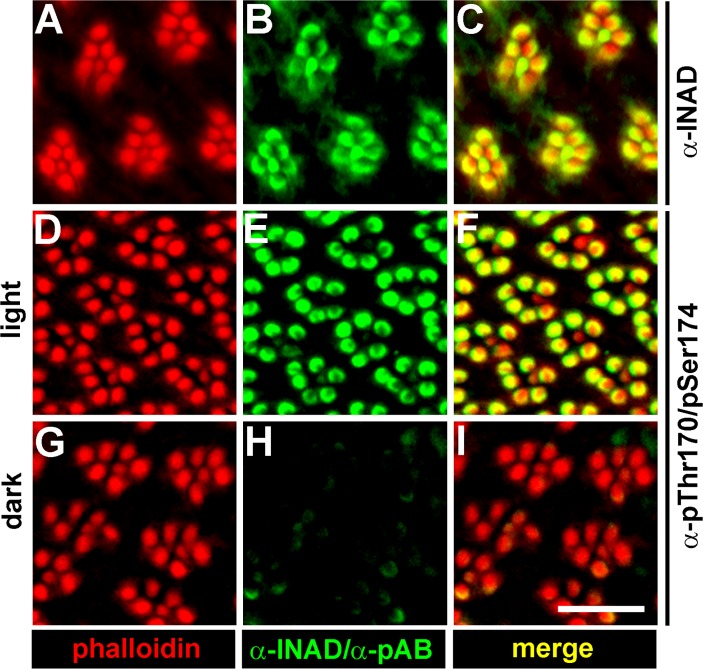
Immunocytochemistry Reveals Rhabdomeral Localization of INAD Phosphorylated at Thr170/Ser174. Tangential cryosections from the eyes of light- or dark-adapted wild type flies were probed with a pan-specific α-INAD antibody or with α-pThr170/pSer174 (green) and with phalloidin that specifically labels the rhabdomeres (red). Colocalizing signals from both channels appear yellow in the merged panels. Scale bar, 10 μm; α-pAB, phosphospecific antibody.

Since the identified INAD phosphorylation sites Thr170 and Ser174 are predicted eye-PKC phosphorylation sites (according to the PPSP tool, http://ppsp.biocuckoo.org), and eye-PKC is a member of the INAD signaling complex [1–4,7], we generated a phosphospecific polyclonal antibody directed against a synthetic phosphopeptide harboring two phosphorylated residues that correspond to pThr170 and pSer174 in INAD. Immunoaffinity purification against the same doubly phosphorylated peptide yielded an antibody population (from here on referred to as α-pThr170/pSer174) that specifically detected the phosphorylated but not a corresponding non-phosphorylated synthetic INAD peptide (S2A Fig.). To rule out possible cross reactivity with other phosphorylated peptides, we tested three additional synthetic phosphopeptides that comprised sequences from the *Drosophila* TRP protein (S2A Fig.). We also confirmed linearity of the signal obtained in Western blots by loading different amounts of phosphorylated INAD from light-adapted fly heads (S2B and C Fig.). Since the phosphorylation pattern of a protein can influence its subcellular localization, we used the phosphospecific antibody to investigate the subcellular localization of INAD phosphorylated at Thr170/Ser174 by immunocytochemistry. We observed strong labeling of the rhabdomeres of photoreceptor cells R1 to R6 in light-adapted flies that colocalized with phalloidin labeling of the rhabdomeres (Fig. 3D-E). Interestingly, no labeling was observed in rhabdomeres of R7 cells. R7 cells express RH3 and RH4, respectively, both of which exhibit absorbance maxima within the ultraviolet range. Presumably, the white light source we used does not emit high enough intensities of short wavelength light to sufficiently activate the UV sensitive rhodopsins RH3 or RH4. In dark-adapted flies, we observed only faint labeling of INAD-pThr170/pSer174 (Fig. 3G-H). Using a pan-specific α-INAD antibody, the rhabdomeres of photoreceptor cells R1 to R6 as well as R7 were labeled (Fig. 3A-C). These findings show that INAD is light-dependently phosphorylated at Thr170/Ser174 and that this phosphorylated form is not a transport form of INAD but, like the majority of INAD, is present in the rhabdomeres (Fig. 3A-C). The phosphorylation of Thr170/Ser174 may thus contribute to the observed light-dependent isoelectric point shift of INAD (see Fig. 1).

### INAD Phosphorylation at Thr170/Ser174 is Triggered by the Phototransduction Cascade

**Fig 4:**
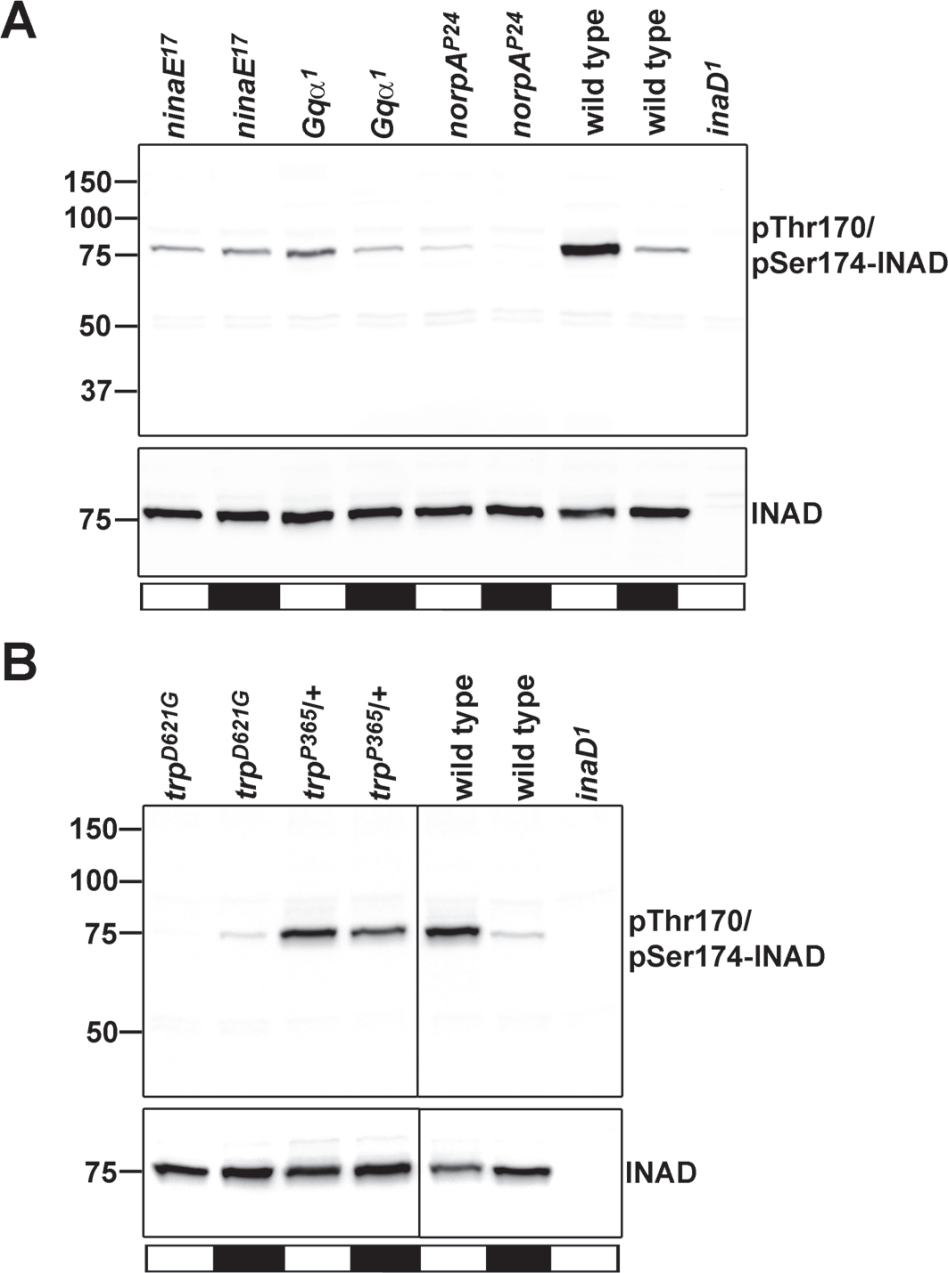
INAD Phosphorylation at Thr170/Ser174 in Different Mutants of the Phototransduction Cascade. Western blot analysis assessing pThr170/pSer174-INAD and INAD regardless of its phosphorylation state in wild type and in the indicated mutants lacking components of the phototransduction cascade (A) or expressing defective alleles of the TRP ion channel (B). Flies were light or dark adapted for 12–18 hours as indicated by the bars below the blots (white, 12–18 h light; black, 12–18 h dark). Molecular mass markers (in kDa) are indicated on the left.

The light-dependent isoelectric point shift of INAD was not observed in the *norpA* ^*P24*^ mutant lacking phospholipase Cβ (see Fig. 1). This finding suggests that phospholipase Cβ and possibly other components of the phototransduction cascade are essential for light-triggered INAD phosphorylation. To test this hypothesis, we performed Western blot analyses using the α-pThr170/pSer174 antibody to investigate the phosphorylation state of Thr170/Ser174 in different mutants of the phototransduction cascade under different light conditions (Fig. 4). As expected, in illuminated wild type flies, we observed a strong signal at 75 kDa whereas in dark-kept wild type flies, we observed a faint signal. In the *inaD* null mutant, *inaD* ^*1*^, no signal was detected. In light-adapted *ninaE* ^*17*^ mutants lacking the major rhodopsin of the fly, RH1, we observed a strongly reduced signal when compared to the wild type. Dark-adapted *ninaE* ^*17*^ flies exhibited a faint signal as was found for the wild type. In the *Gqα* ^*1*^ hypomorphic mutant exhibiting an approximately 1000fold reduction of the visual response [22], we also observed a reduction in Thr170/Ser174 phosphorylation in light-adapted flies. However, phosphorylation of Thr170/Ser174 was stronger in light-adapted *Gqα* ^*1*^ flies than in dark-adapted *Gqα* ^*1*^ flies which may be attributed to the residual light response of this mutant. In the *norpA* ^*P24*^ mutant lacking phospholipase Cβ, we observed a drastic reduction in phosphorylation of Thr170/Ser174. These results show that the phototransduction cascade is essential to trigger phosphorylation of Thr170/Ser174. The last step of the phototransduction cascade is the opening of the TRP and TRPL channels. The TRP channel is permeable mostly for Ca^2+^ ions. Since the intracellular Ca^2+^ level is a major signal to regulate protein kinase C activity and Thr170 and Ser174 are predicted to be PKC phosphorylation sites, we wondered whether the light-induced increase in the intracellular Ca^2+^ level regulates phosphorylation of Thr170 and Ser174. To answer that question, we utilized two available *trp* mutants, *trp* ^*D621G*^ and *trp* ^*P365*^. The *trp* ^*D621G*^ fly transgenically expresses a TRP channel with a pore that is impermeable for Ca^2+^ resulting in low intracellular Ca^2+^ levels [25]. The *trp* ^*P365*^ mutant expresses a TRP channel that is constitutively open resulting in permanent Ca^2+^ permeability and high intracellular Ca^2+^ levels [20]. As a result, we found weak phosphorylation of Thr170/Ser174 in the *trp* ^*D621G*^ transgenic fly and strong phosphorylation in the *trp* ^*P365*^ mutant regardless of the light conditions. Taken together, these results propose that an elevation of intracellular Ca^2+^ is sufficient to trigger INAD phosphorylation at Thr170/Ser174.

### Eye-PKC is Involved in INAD Phosphorylation

**Fig 5:**
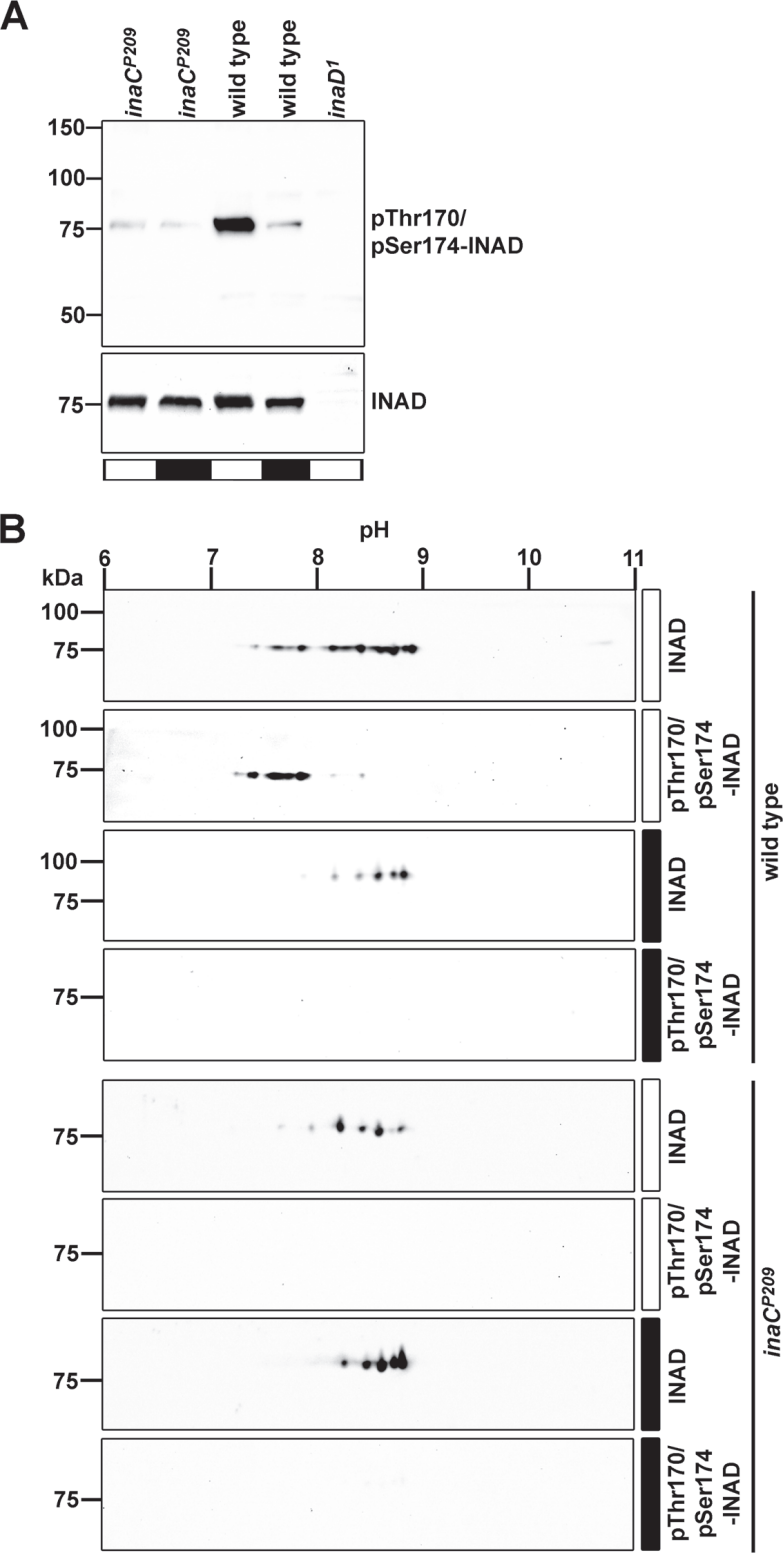
INAD Phosphorylation Patterns in the eye-PKC Null Mutant *inaC* ^*P209*^. A, Western blot analysis assessing pThr170/pSer174-INAD and INAD in wild type and *inaC* ^*P209*^ mutant flies. Flies were light (white bar) or dark adapted (black bar) for 12–18 h. B, 2D Western blot analysis assessing pThr170/pSer174-INAD and INAD in wild type flies and *inaC* ^*P209*^ mutant flies. Flies were light or dark adapted for 12–18 hours as indicated by the bars beside the blots (white, 12–18 h light; black, 12–18 h dark). Molecular mass markers (in kDa) are indicated to the left, pH range to the top.

A likely candidate for the kinase phosphorylating INAD at Thr170/Ser174 is eye-PKC because eye-PKC is a Ca^2+^-dependent kinase that is attached to INAD and has previously been shown to phosphorylate INAD *in vitro* [29,30]. Additionally, both phosphorylation sites are embedded within a PKC consensus sequence. We therefore analyzed the phosphorylation state of INAD at Thr170/Ser174 in the eye-PKC null mutant *inaC* ^*P209*^ using our phosphospecific antibody in immunoblot analyses. As a result, phosphorylation of Thr170/Ser174 was drastically reduced in light-adapted *inaC* ^*P209*^ mutants as compared to light-adapted wild type flies (Fig. 5A). Thus, eye-PKC is essentially needed for the light-dependent phosphorylation of Thr170/Ser174. We then probed immunoblots of two-dimensionally separated head extracts from light- and dark-adapted wild type and *inaC* ^*P209*^ mutant flies with an α-INAD antibody that detected INAD regardless of its phosphorylation state, and with the α-pThr170/pSer174 antibody (Fig. 5B). Using the α-INAD antibody, we detected striking differences in the spot patterns of light-adapted *inaC* ^*P209*^ mutant and wild type flies. Obviously, phosphorylation in light-adapted *inaC* ^*P209*^ mutant flies was compromised since spots located in more acidic pH ranges were not as prominent as the ones that were obtained from wild type head extracts (compare the 1^st^ and 5^th^ panel of Fig. 5B). Spot patterns were similar in dark-adapted *inaC* ^*P209*^ and wild type flies (compare the 3^rd^ and 7^th^ panel of Fig. 5B). Additionally, the α-pThr170/pSer174 antibody detected spots in the more acidic pH range only in light-adapted wild type flies but yielded no signal in light-adapted *inaC* ^*P209*^ flies. However, in light-adapted *inaC* ^*P209*^ flies, a reduction of spots in the basic range and a gain of spots in the more acidic range was still obvious, when compared to dark-adapted *inaC* ^*P209*^ flies. This latter finding suggests that besides eye-PKC, other protein kinases might contribute to the light-dependent phosphorylation of some INAD sites. Using the α-pThr170/pSer174 antibody, in light-adapted wild type flies (Fig. 5B, 2^nd^ panel), only spots in the more acidic range were detected whereas no spots were detected in extracts from dark-adapted wild type flies. In the *inaC* ^*P209*^ mutant, no spots were detected using the α-pThr170/pSer174 antibody regardless of the light condition. The fact that more than two spots were detected with the α-pThr170/pSer174 antibody in extracts from light-adapted flies points to the existence of different INAD isoforms that are phosphorylated at Thr170/Ser174 and at several other sites.

## Discussion

In this study, we report the identification of eight INAD phosphorylation sites by a nano-HPLC-MS approach. INAD phosphorylation was further investigated by 1D or 2D gel electrophoresis and concomitant immunoblotting using a pan-specific α-INAD antibody detecting INAD regardless of its phosphorylation state and a phosphospecific antibody that detected phosphorylation of INAD at T170/S174. Comparison of the intensity of spots on the 2D immunoblots suggests that a considerable amount of INAD becomes light-dependently phosphorylated supporting a physiological relevance of INAD phosphorylation. After light adaptation, about half of the INAD spots on 2D gels are detected by an α-T170/S174 antibody, suggesting that a significant number of the INAD molecules are phosphorylated at one of these two sites in the light. We found that the light-dependent isoelectric point shift observed in 2D blots depended on PLCβ indicating an involvement of the phototransduction cascade in INAD phosphorylation. Indeed, impairments in rhodopsin, Gqα, and PLCβ resulted in compromised INAD phosphorylation at Thr170/Ser174. Manipulation of intracellular Ca^2+^ concentrations by usage of different *trp* mutants resulted in altered phosphorylation levels of Thr170/Ser174. Elevated Ca^2+^ levels led to strong phosphorylation whereas lowered Ca^2+^ levels resulted in weak phosphorylation of T170/S174. We propose that INAD phosphorylation at Thr170/Ser174 is ultimately regulated by intracellular Ca^2+^ levels. Upon phosphatase treatment of fly head protein extracts, INAD spots on 2D blots became even more condensed than in dark-adapted untreated head extracts. This points to the existence of sites that are phosphorylated in the dark. Indeed, phosphorylated Ser40 was identified from dark-adapted flies in our mass spectrometry approach. However, we cannot rule out that there is also Ser40 phosphorylation or even enhanced Ser40 phosphorylation in the light. Generally, we were not able to detect INAD phosphorylation sites robustly enough for quantification by mass spectrometry.

Thr170 and Ser174 are predicted PKC phosphorylation sites and PKC is activated by rising intracellular Ca^2+^ levels and by DAG. Indeed, in the eye-PKC null mutant, *inaC* ^*P209*^, phosphorylation of INAD at Thr170/Ser174 was drastically reduced.

This finding is in line with *in vitro* studies showing that INAD can be phosphorylated in purified INAD signaling complexes containing eye-PKC [29,30]. The finding that INAD becomes phosphorylated at Thr170/Ser174 in the rhabdomeres is also in line with the finding that phosphorylation of these sites depends on eye-PKC. Since eye-PKC is a member of the INAD signaling complex, it is localized to the rhabdomeres. However, not all INAD phosphorylation sites identified in this study were eye-PKC-dependent. First, the Ser40 phosphopeptide was identified from dark-adapted eye-PKC null mutant flies. Second, the absence of eye-PKC did not result in a complete lack of the light-dependent isoelectric point shift of INAD isoforms in 2D blots. We conclude that additional kinases are involved in INAD phosphorylation. A possible candidate for such a protein kinase is PKC53E, a PKC that is also expressed in the eye and was previously shown to act redundantly with eye-PKC in phosphorylation of the TRP phosphorylation site T849 [27].

Putative physiological effects of INAD phosphorylation might at least in part be conveyed by the regulation of the formation of the disulfide bond within PDZ5. As was shown, formation of the disulfide bond depends on eyePKC [16]. Since disruption of the hydrogen bond between PDZ4 and PDZ5 resulted in a dramatic increase in phosphorylation of a heterologously-expressed PDZ45 fragment [17], phosphorylation is enhanced when PDZ4 and PDZ5 are uncoupled. Reversely, phosphorylation might enhance uncoupling of PDZ4 and PDZ5 and thus formation of the disulfide bond disrupting the binding pocket for TRP. The only *in vivo* phosphorylation site that we detected in the PDZ4/PDZ5 region was T544. However, the available data do not allow us to estimate the phosphate occupancy of this site in light- and dark-adapted flies. It remains to be determined whether or not phosphorylation of T544 affects INAD structure and is physiologically relevant. Additionally, Ser163, Thr170, and Ser174 are located in the linker region connecting PDZ1 and PDZ2 that harbors a putative calmodulin binding site [9]. More specifically, calmodulin bound to a heterologously-expressed INAD fragment spanning residues 146 to 235 [9]. Binding of calmodulin to INAD *in vivo* may be modulated by phosphorylation of Ser163, Thr170, and Ser174. In this study we demonstrate that INAD is not phosphorylated at Thr170 and Ser174 in the dark while a significant amount of INAD molecules, represented by distinct spots on 2D gels, becomes phosphorylated at these sites in the light.

## Supporting Information

S1 Fig Fragmentation Spectra of INAD Phosphopeptides. Figs. A to G show fragmentation spectra and tables derived from tryptic INAD peptides. The coverage of the peptide sequence by b and y ions and the calculated mass for each fragment ion are shown in the tables below the spectra, in which observed b and y ions are highlighted in red and blue, respectively.
(DOCX)

S2 Fig Validation of Antibodies. A, 2.5 μg of phospho- and dephosphopeptides were spotted onto a nitrocellulose membrane and the membrane was then blocked and incubated with the α-pThr170/pSer174 antibody and a secondary α-rabbit IgG conjugated to horse radish peroxidase. Enhanced chemiluminescence signals were recorded. Phosphopeptides were NH_2_-CGRKK(pT)QKGD-CONH_2_ containing phosphorylated Thr849 of TRP, NH2-CARKN(pT)FASD-CONH2 containing phosphorylated Thr864 of TRP, NH2-CADEVpSLADD-CONH2 containing phosphorylated Ser936 of TRP, and NH2-T(pT)FTA(pS)MRQC-CONH2 containing phosphorylated Thr170 and Ser174 of INAD. The dephosphopeptides were similar to the respective phosphopeptides except for the lack of the phosphoryl groups. B and C, To check linearity of the signal intensities obtained with the antibodies, different amounts of protein extracts from wild type heads were supplemented with protein extracts from *inaD* ^*1*^ null mutant heads to ensure equal overall protein content. Three head equivalents were loaded onto a gel and subjected to Western blot analysis using α-INAD and α-pThr170/pSer174 antibodies. B shows representative Western blots and C shows results from three independent experiments. Error bars show SEM.
(DOCX)
