# Supplementary material for: Light-Dependent Phosphorylation of the Drosophila Inactivation No Afterpotential D (INAD) Scaffolding Protein at Thr170 and Ser174 by Eye-Specific Protein Kinase C
Source: PLoS One. 2015 Mar 23;10(3):e0122039. doi: 10.1371/journal.pone.0122039 (PMC4370639; doi:10.1371/journal.pone.0122039)
Supplement: S1 Fig — Figs. A to G show fragmentation spectra and tables derived from tryptic INAD peptides. The coverage of the peptide sequence by b and y ions and the calculated mass for each fragment ion are shown in the tables below the spectra, in which observed b and y ions are highlighted in red and blue, respectively. (DOCX) [file pone.0122039.s001.docx]

**A**

pT10

pyroQGpTAGELIHMVTLDK

m/z = 838.39 (+2)


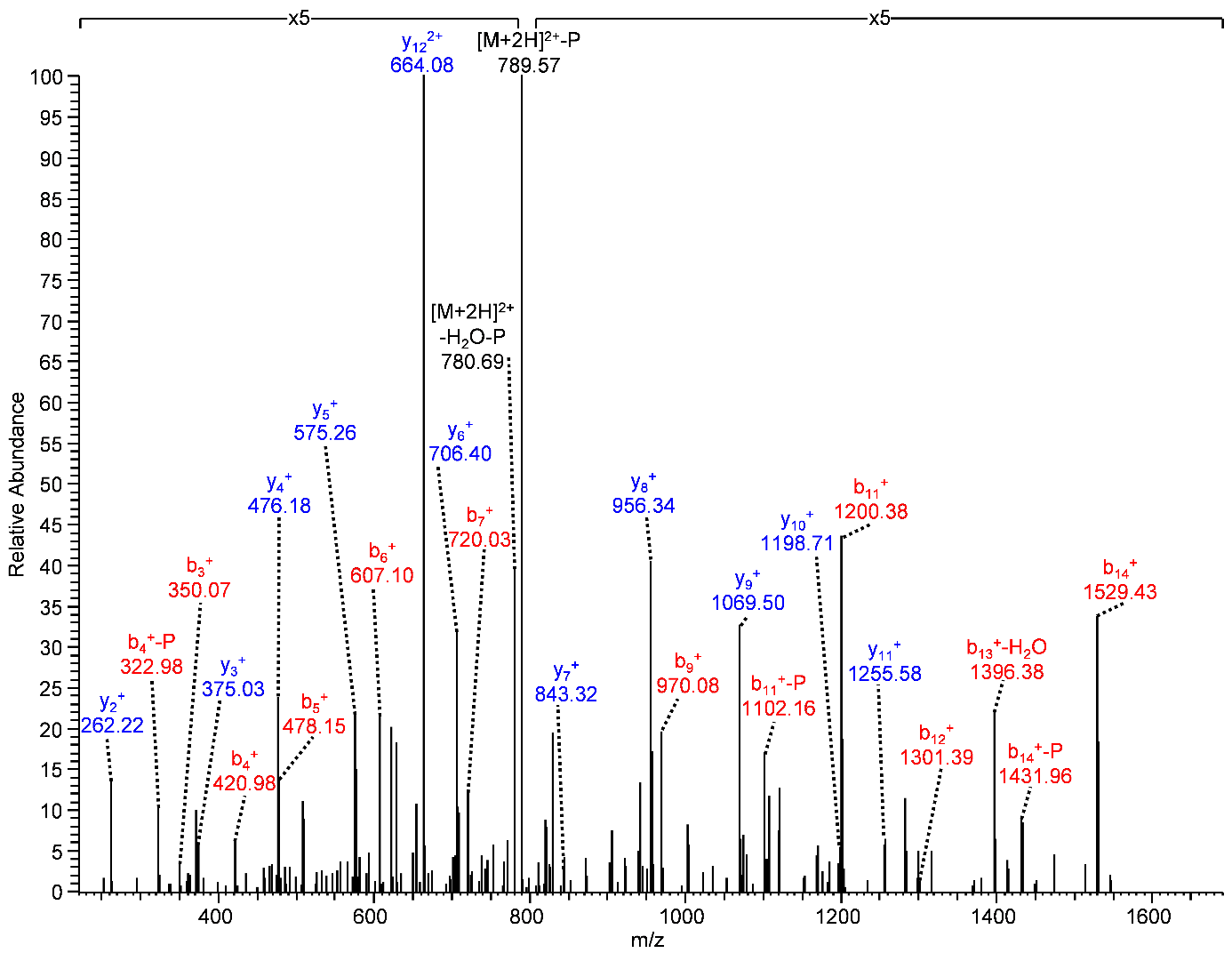


| **#1** | **b⁺** | **b²⁺** | **Seq.** | **y⁺** | **y²⁺** | **#2** |
| --- | --- | --- | --- | --- | --- | --- |
| 1 | 112.03931 | 56.52329 | Q-Gln->pyro-Glu |  |  | 15 |
| 2 | 169.06078 | 85.03403 | G | 1564.74415 | 782.87571 | 14 |
| 3 | 350.07479 | 175.54103 | T-Phospho | 1507.72268 | 754.36498 | 13 |
| 4 | 421.11191 | 211.05959 | A | 1326.70867 | 663.85797 | 12 |
| 5 | 478.13338 | 239.57033 | G | 1255.67155 | 628.33941 | 11 |
| 6 | 607.17598 | 304.09163 | E | 1198.65008 | 599.82868 | 10 |
| 7 | 720.26005 | 360.63366 | L | 1069.60748 | 535.30738 | 9 |
| 8 | 833.34412 | 417.17570 | I | 956.52341 | 478.76534 | 8 |
| 9 | 970.40303 | 485.70515 | H | 843.43934 | 422.22331 | 7 |
| 10 | 1101.44353 | 551.22540 | M | 706.38043 | 353.69385 | 6 |
| 11 | 1200.51195 | 600.75961 | V | 575.33993 | 288.17360 | 5 |
| 12 | 1301.55963 | 651.28345 | T | 476.27151 | 238.63939 | 4 |
| 13 | 1414.64370 | 707.82549 | L | 375.22383 | 188.11555 | 3 |
| 14 | 1529.67065 | 765.33896 | D | 262.13976 | 131.57352 | 2 |
| 15 |  |  | K | 147.11281 | 74.06004 | 1 |

**B**

pS40

GEVKDpSPNTK

m/z = 577.76 (+2)


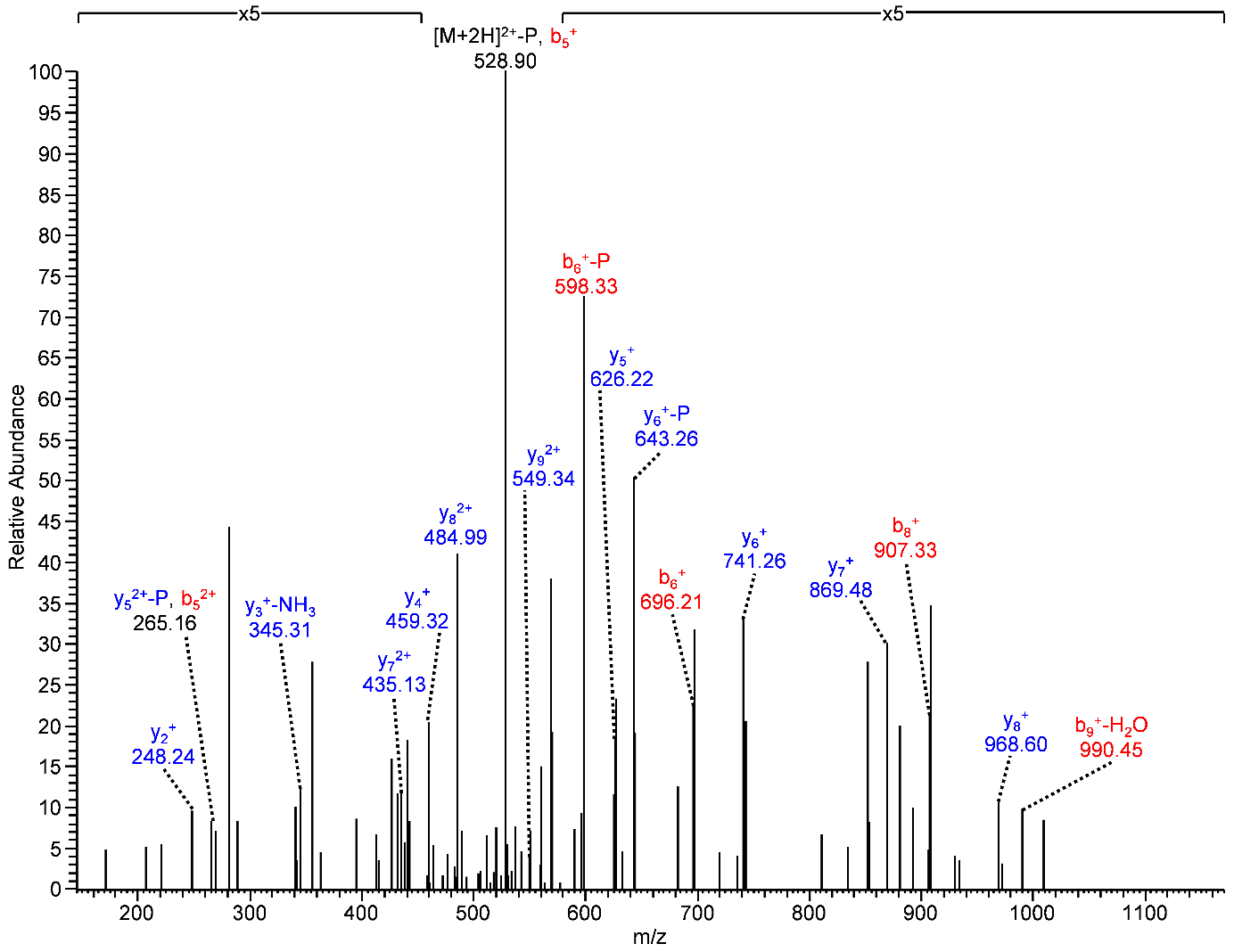


| **#1** | **b⁺** | **b²⁺** | **Seq.** | **y⁺** | **y²⁺** | **#2** |
| --- | --- | --- | --- | --- | --- | --- |
| 1 | 58.02875 | 29.51801 | G |  |  | 10 |
| 2 | 187.07135 | 94.03931 | E | 1097.48749 | 549.24738 | 9 |
| 3 | 286.13977 | 143.57352 | V | 968.44489 | 484.72608 | 8 |
| 4 | 414.23474 | 207.62101 | K | 869.37647 | 435.19187 | 7 |
| 5 | 529.26169 | 265.13448 | D | 741.28150 | 371.14439 | 6 |
| 6 | 696.26005 | 348.63366 | S-Phospho | 626.25455 | 313.63091 | 5 |
| 7 | 793.31282 | 397.16005 | P | 459.25619 | 230.13173 | 4 |
| 8 | 907.35575 | 454.18151 | N | 362.20342 | 181.60535 | 3 |
| 9 | 1008.40343 | 504.70535 | T | 248.16049 | 124.58388 | 2 |
| 10 |  |  | K | 147.11281 | 74.06004 | 1 |

**C**

pS163

pyroQQpSMQKR

484.70 (+2)


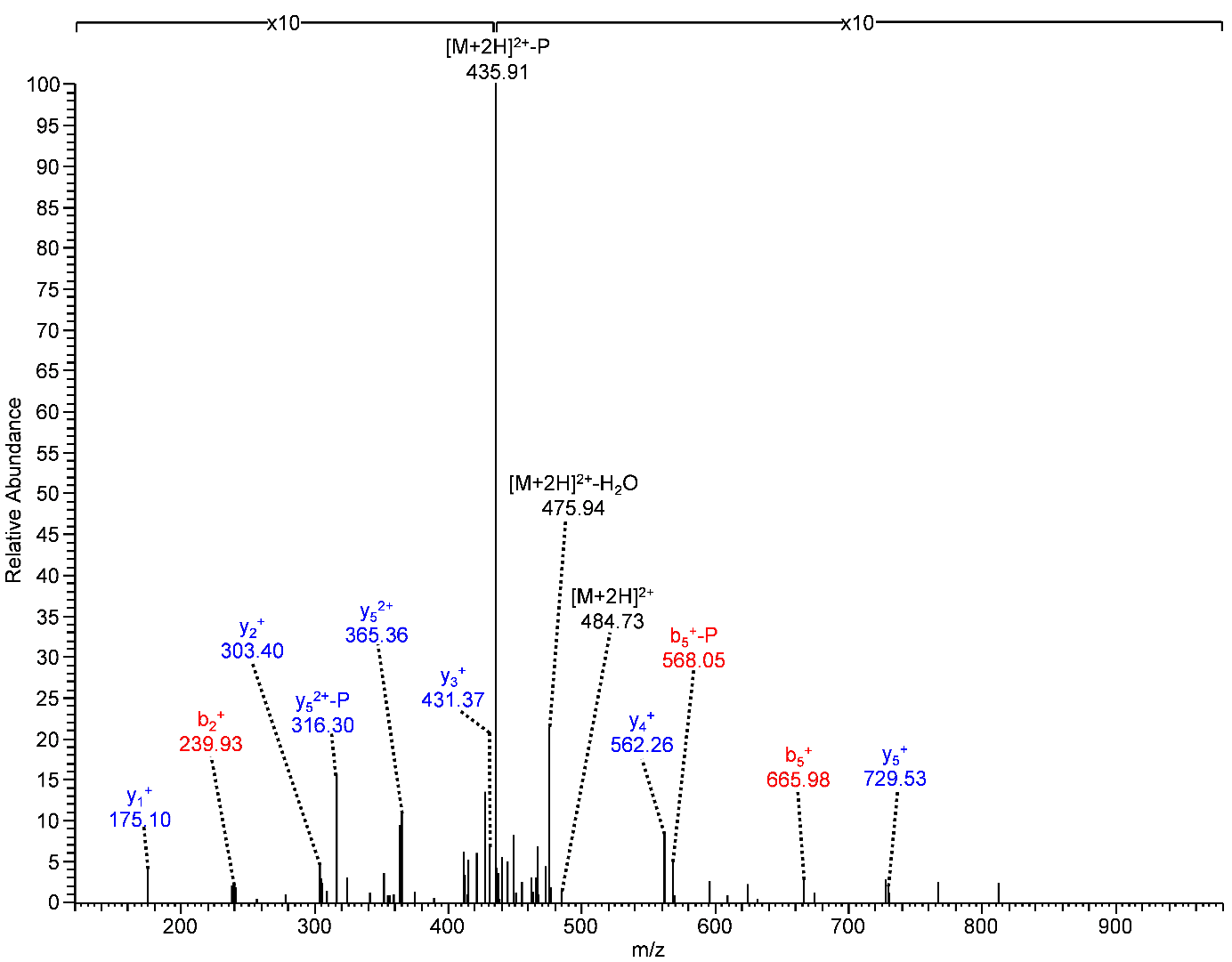


| **#1** | **b⁺** | **b²⁺** | **Seq.** | **y⁺** | **y²⁺** | **#2** |
| --- | --- | --- | --- | --- | --- | --- |
| 1 | 112.03931 | 56.52329 | Q-Gln->pyro-Glu |  |  | 7 |
| 2 | 240.09789 | 120.55258 | Q | 857.36995 | 429.18861 | 6 |
| 3 | 407.09625 | 204.05176 | S-Phospho | 729.31137 | 365.15932 | 5 |
| 4 | 538.13675 | 269.57201 | M | 562.31301 | 281.66014 | 4 |
| 5 | 666.19533 | 333.60130 | Q | 431.27251 | 216.13989 | 3 |
| 6 | 794.29030 | 397.64879 | K | 303.21393 | 152.11060 | 2 |
| 7 |  |  | R | 175.11896 | 88.06312 | 1 |

**D**

pT170/pS174

NTTFpTApSMR

m/z = 594.71 (+2)


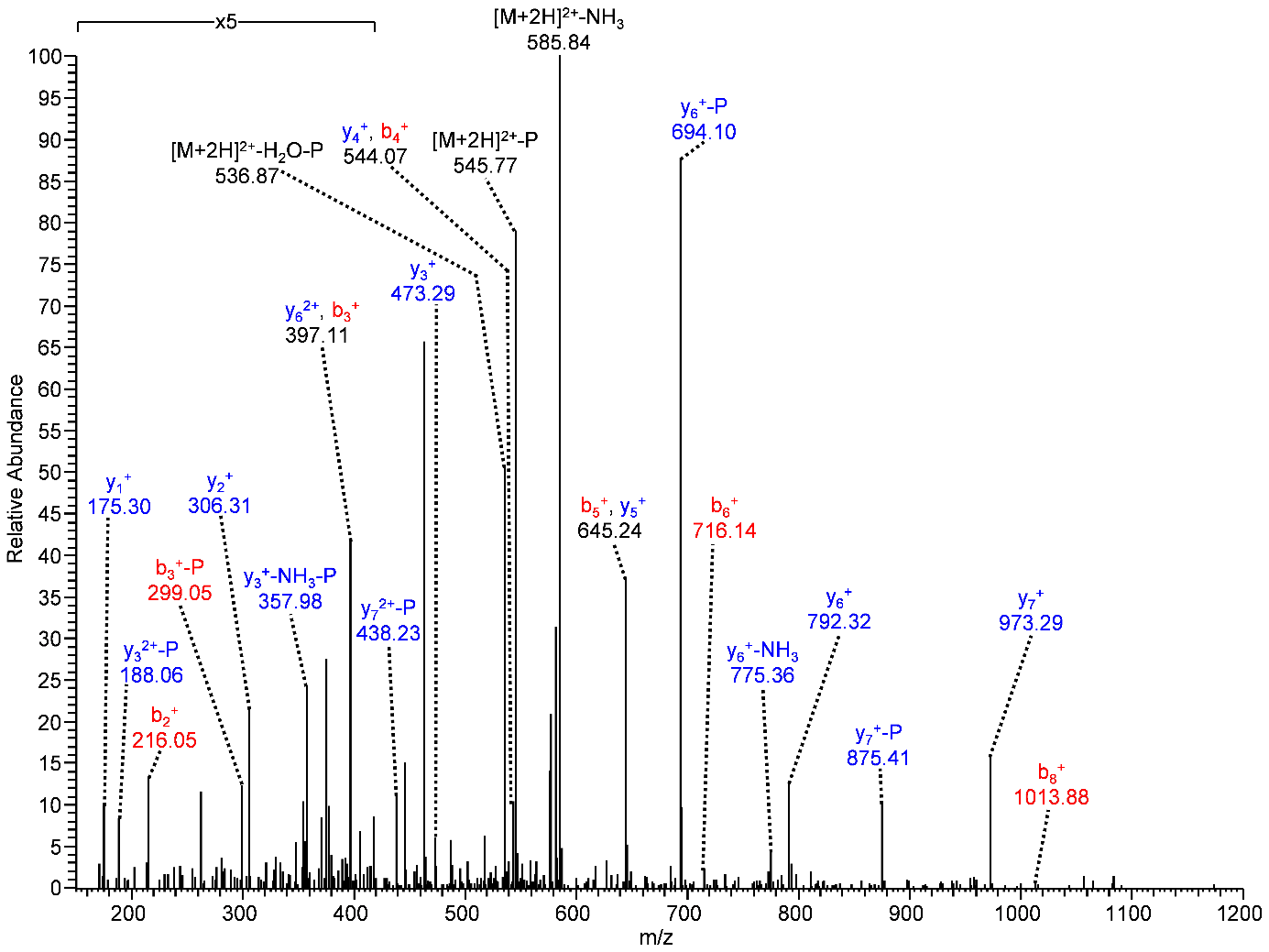


| **#1** | **b⁺** | **b²⁺** | **Seq.** | **y⁺** | **y²⁺** | **#2** |
| --- | --- | --- | --- | --- | --- | --- |
| 1 | 115.05021 | 58.02874 | N |  |  | 9 |
| 2 | 216.09789 | 108.55258 | T | 1074.37273 | 537.69000 | 8 |
| 3 | 397.11190 | 199.05959 | T-Phospho | 973.32505 | 487.16616 | 7 |
| 4 | 544.18032 | 272.59380 | F | 792.31104 | 396.65916 | 6 |
| 5 | 645.22800 | 323.11764 | T | 645.24262 | 323.12495 | 5 |
| 6 | 716.26512 | 358.63620 | A | 544.19494 | 272.60111 | 4 |
| 7 | 883.26348 | 442.13538 | S-Phospho | 473.15782 | 237.08255 | 3 |
| 8 | 1014.30398 | 507.65563 | M | 306.15946 | 153.58337 | 2 |
| 9 |  |  | R | 175.11896 | 88.06312 | 1 |

**E**

pS257

DApSKPLGLALAGHK

m/z = 486.59 (+3)


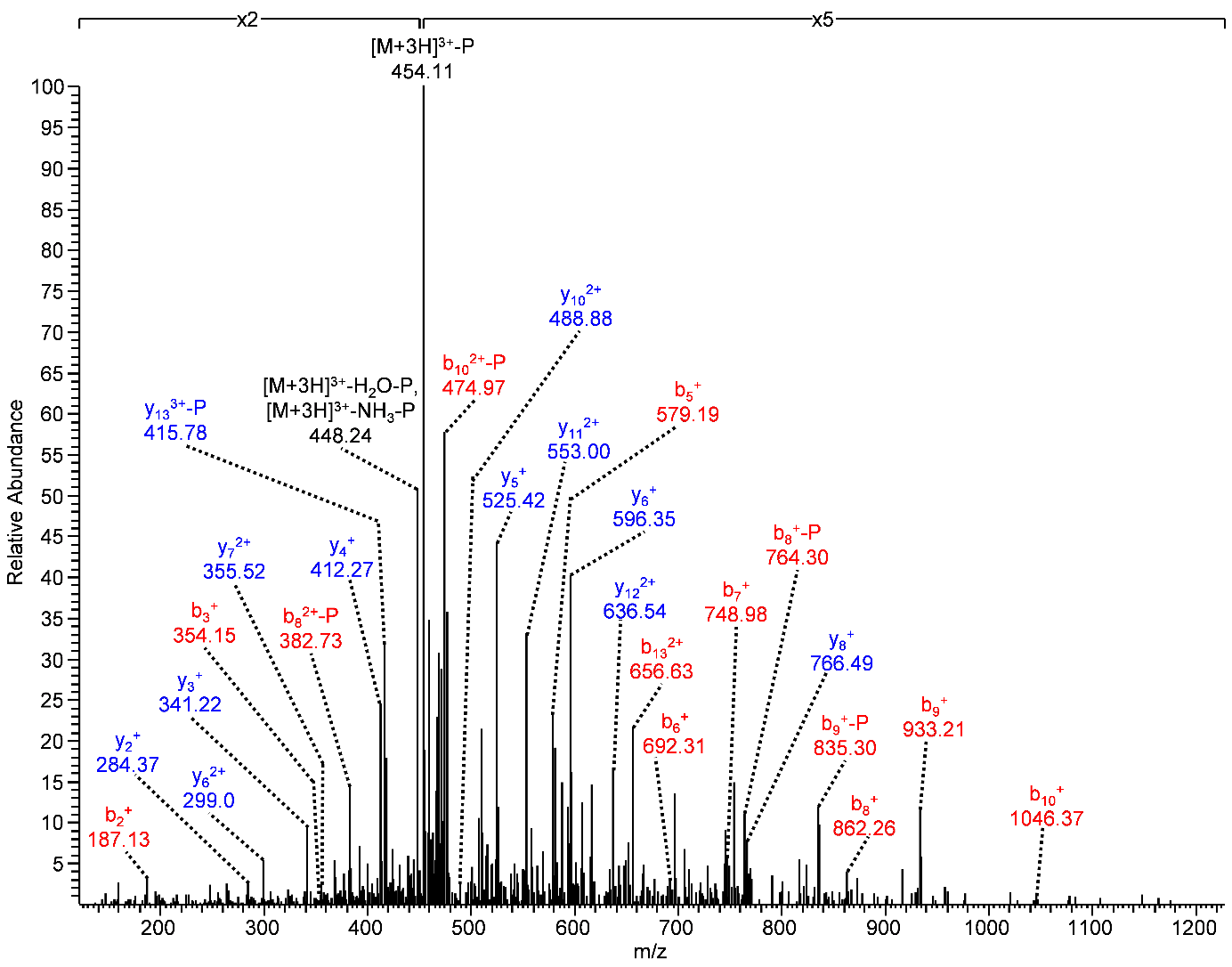


| **#1** | **b⁺** | **b²⁺** | **Seq.** | **y⁺** | **y²⁺** | **#2** |
| --- | --- | --- | --- | --- | --- | --- |
| 1 | 116.03423 | 58.52075 | D |  |  | 14 |
| 2 | 187.07135 | 94.03931 | A | 1342.72433 | 671.86580 | 13 |
| 3 | 354.06971 | 177.53849 | S-Phospho | 1271.68721 | 636.34724 | 12 |
| 4 | 482.16468 | 241.58598 | K | 1104.68885 | 552.84806 | 11 |
| 5 | 579.21745 | 290.11236 | P | 976.59388 | 488.80058 | 10 |
| 6 | 692.30152 | 346.65440 | L | 879.54111 | 440.27419 | 9 |
| 7 | 749.32299 | 375.16513 | G | 766.45704 | 383.73216 | 8 |
| 8 | 862.40706 | 431.70717 | L | 709.43557 | 355.22142 | 7 |
| 9 | 933.44418 | 467.22573 | A | 596.35150 | 298.67939 | 6 |
| 10 | 1046.52825 | 523.76776 | L | 525.31438 | 263.16083 | 5 |
| 11 | 1117.56537 | 559.28632 | A | 412.23031 | 206.61879 | 4 |
| 12 | 1174.58684 | 587.79706 | G | 341.19319 | 171.10023 | 3 |
| 13 | 1311.64575 | 656.32651 | H | 284.17172 | 142.58950 | 2 |
| 14 |  |  | K | 147.11281 | 74.06004 | 1 |

**F**

pT349

KFPpTASDETK

m/z = 602.27 (+2)


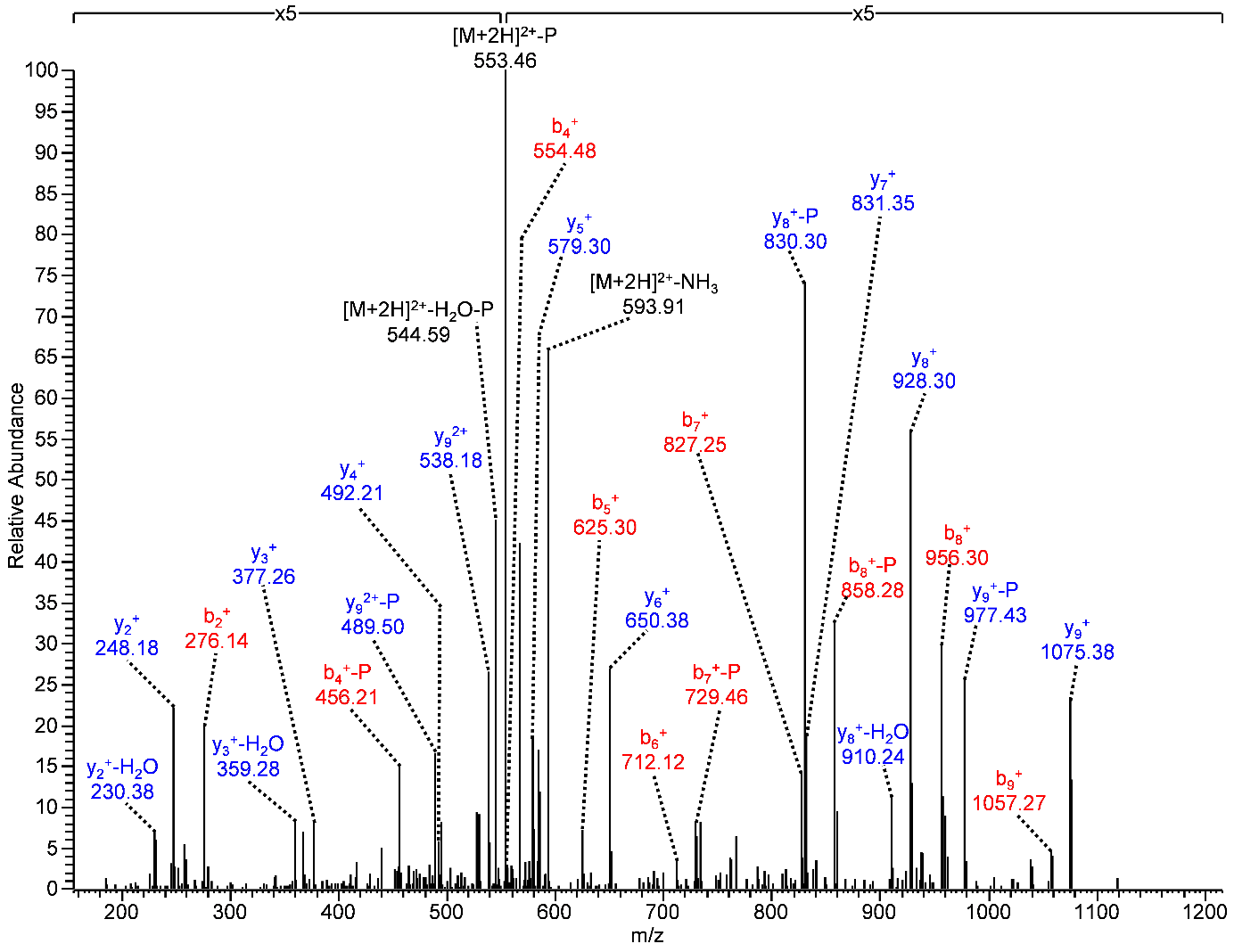


| **#1** | **b⁺** | **b²⁺** | **Seq.** | **y⁺** | **y²⁺** | **#2** |
| --- | --- | --- | --- | --- | --- | --- |
| 1 | 129.10225 | 65.05476 | K |  |  | 10 |
| 2 | 276.17067 | 138.58897 | F | 1075.43439 | 538.22083 | 9 |
| 3 | 373.22344 | 187.11536 | P | 928.36597 | 464.68662 | 8 |
| 4 | 554.23745 | 277.62236 | T-Phospho | 831.31320 | 416.16024 | 7 |
| 5 | 625.27457 | 313.14092 | A | 650.29919 | 325.65323 | 6 |
| 6 | 712.30660 | 356.65694 | S | 579.26207 | 290.13467 | 5 |
| 7 | 827.33355 | 414.17041 | D | 492.23004 | 246.61866 | 4 |
| 8 | 956.37615 | 478.69171 | E | 377.20309 | 189.10518 | 3 |
| 9 | 1057.42383 | 529.21555 | T | 248.16049 | 124.58388 | 2 |
| 10 |  |  | K | 147.11281 | 74.06004 | 1 |

**G**

pT544

IFDHICDINGpTPIHVGSMTTLK

m/z = 850.40 (+3)


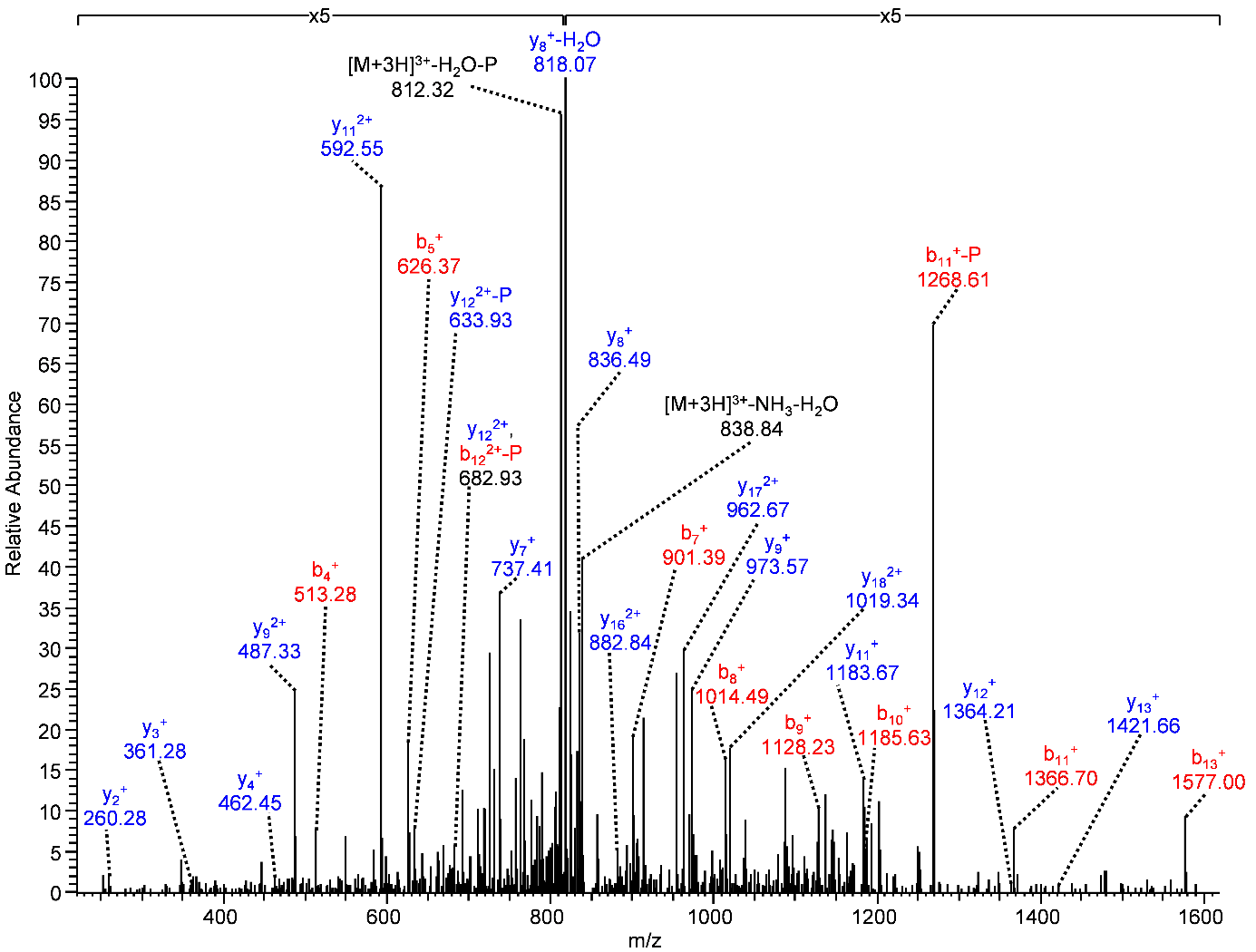


| **#1** | **b⁺** | **b²⁺** | **Seq.** | **y⁺** | **y²⁺** | **#2** |
| --- | --- | --- | --- | --- | --- | --- |
| 1 | 114.09135 | 57.54931 | I |  |  | 22 |
| 2 | 261.15977 | 131.08352 | F | 2436.10885 | 1218.55806 | 21 |
| 3 | 376.18672 | 188.59700 | D | 2289.04043 | 1145.02385 | 20 |
| 4 | 513.24563 | 257.12645 | H | 2174.01348 | 1087.51038 | 19 |
| 5 | 626.32970 | 313.66849 | I | 2036.95457 | 1018.98092 | 18 |
| 6 | 786.36035 | 393.68381 | C-Carbamidomethyl | 1923.87050 | 962.43889 | 17 |
| 7 | 901.38730 | 451.19729 | D | 1763.83984 | 882.42356 | 16 |
| 8 | 1014.47137 | 507.73932 | I | 1648.81289 | 824.91008 | 15 |
| 9 | 1128.51430 | 564.76079 | N | 1535.72882 | 768.36805 | 14 |
| 10 | 1185.53577 | 593.27152 | G | 1421.68589 | 711.34658 | 13 |
| 11 | 1366.54978 | 683.77853 | T-Phospho | 1364.66442 | 682.83585 | 12 |
| 12 | 1463.60255 | 732.30491 | P | 1183.65041 | 592.32884 | 11 |
| 13 | 1576.68662 | 788.84695 | I | 1086.59764 | 543.80246 | 10 |
| 14 | 1713.74553 | 857.37640 | H | 973.51357 | 487.26042 | 9 |
| 15 | 1812.81395 | 906.91061 | V | 836.45466 | 418.73097 | 8 |
| 16 | 1869.83542 | 935.42135 | G | 737.38624 | 369.19676 | 7 |
| 17 | 1956.86745 | 978.93736 | S | 680.36477 | 340.68602 | 6 |
| 18 | 2087.90795 | 1044.45761 | M | 593.33274 | 297.17001 | 5 |
| 19 | 2188.95563 | 1094.98145 | T | 462.29224 | 231.64976 | 4 |
| 20 | 2290.00331 | 1145.50529 | T | 361.24456 | 181.12592 | 3 |
| 21 | 2403.08738 | 1202.04733 | L | 260.19688 | 130.60208 | 2 |
| 22 |  |  | K | 147.11281 | 74.06004 | 1 |
