# Supplementary figures and images for: Light-Dependent Phosphorylation of the Drosophila Inactivation No Afterpotential D (INAD) Scaffolding Protein at Thr170 and Ser174 by Eye-Specific Protein Kinase C
Source: PLoS One. 2015 Mar 23;10(3):e0122039. doi: 10.1371/journal.pone.0122039 (PMC4370639; doi:10.1371/journal.pone.0122039)

­
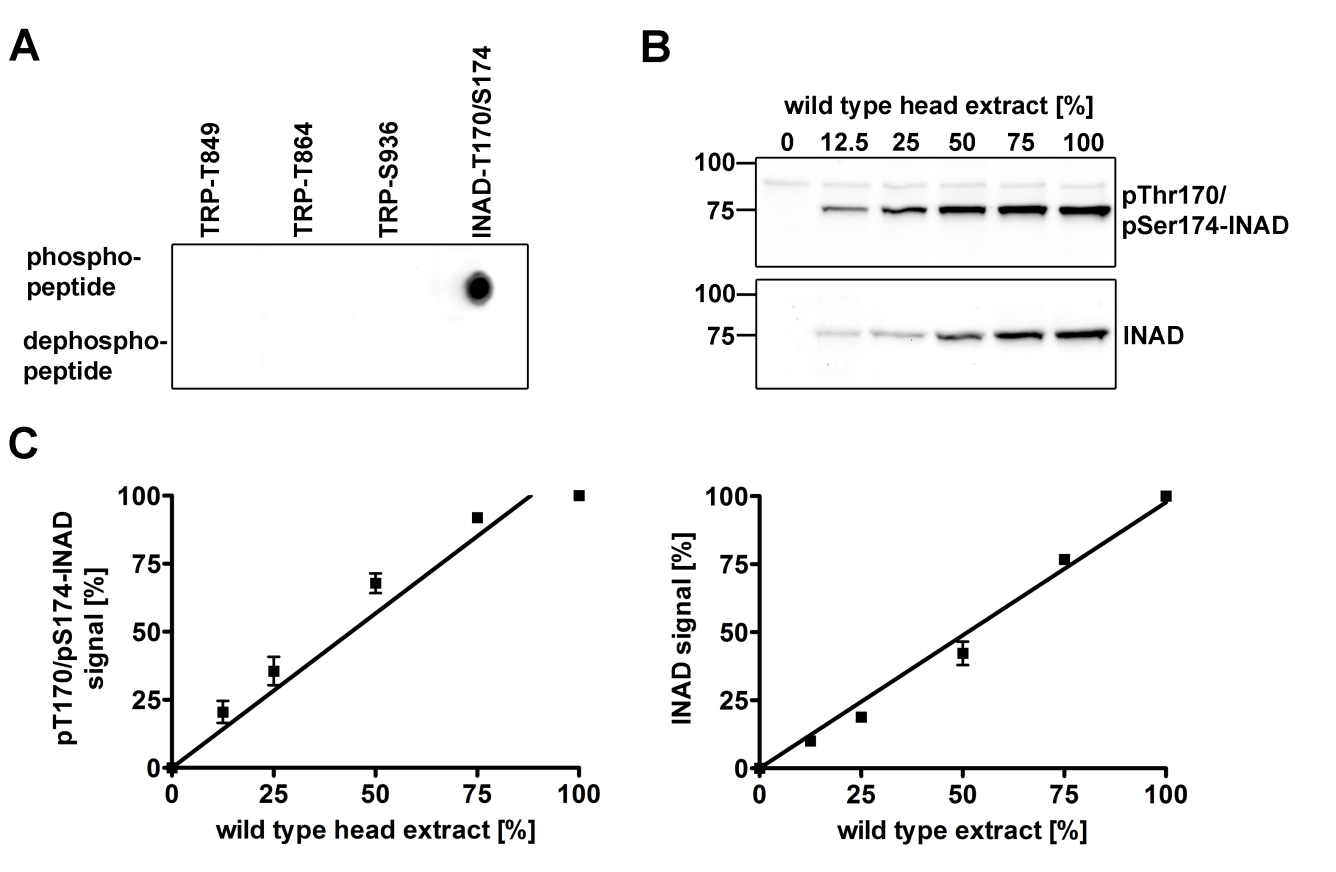

Supplement: S2 Fig — A, 2.5 μg of phospho- and dephosphopeptides were spotted onto a nitrocellulose membrane and the membrane was then blocked and incubated with the α-pThr170/pSer174 antibody and a secondary α-rabbit IgG conjugated to horse radish peroxidase. Enhanced chemiluminescence signals were recorded. Phosphopeptides were NH2-CGRKK(pT)QKGD-CONH2 containing phosphorylated Thr849 of TRP, NH2-CARKN(pT)FASD-CONH2 containing phosphorylated Thr864 of TRP, NH2-CADEVpSLADD-CONH2 containing phosphorylated Ser936 of TRP, and NH2-T(pT)FTA(pS)MRQC-CONH2 containing phosphorylated Thr170 and Ser174 of INAD. The dephosphopeptides were similar to the respective phosphopeptides except for the lack of the phosphoryl groups. B and C, To check linearity of the signal intensities obtained with the antibodies, different amounts of protein extracts from wild type heads were supplemented with protein extracts from inaD 1 null mutant heads to ensure equal overall protein content. Three head equivalents were loaded onto a gel and subjected to Western blot analysis using α-INAD and α-pThr170/pSer174 antibodies. B shows representative Western blots and C shows results from three independent experiments. Error bars show SEM. (DOCX) [file pone.0122039.s002.docx]
